# A Study on Using the Additive Manufacturing Process for the Development of a Closed Pump Impeller for Mechanically Pumped Fluid Loop Systems

**DOI:** 10.3390/ma14040967

**Published:** 2021-02-18

**Authors:** Alexandra Adiaconitei, Ionut Sebastian Vintila, Radu Mihalache, Alexandru Paraschiv, Tiberius Frigioescu, Mihai Vladut, Laurent Pambaguian

**Affiliations:** 1 Satellites and Space Equipment Department, Romanian Research and Development Institute for Gas Turbines (COMOTI), 061126 Bucharest, Romania; sebastian.vintila@comoti.ro (I.S.V.); radu.mihalache@comoti.ro (R.M.); 2 Gas Turbine Special Equipment, Physics and Mechanical Testing Laboratory, Romanian Research and Development Institute for Gas Turbines (COMOTI), 061126 Bucharest, Romania; alexandru.paraschiv@comoti.ro (A.P.); tiberius.frigioescu@comoti.ro (T.F.); 3 Quality Control Technology Department, Romanian Research and Development Institute for Gas Turbines (COMOTI), 061126 Bucharest, Romania; mihai.vladut@comoti.ro; 4 European Space Research and Technology Centre (ESA-ESTEC), Mechanical Department, European Space Agency, 2200 AG Noordwijk, The Netherlands; laurent.pambaguian@esa.int

**Keywords:** additive manufacturing, closed impeller, selective laser melting, surface quality, dimensional accuracy, abrasive flow machining, centrifugal pump, mechanical pump fluid loop

## Abstract

The efficiency of a centrifugal pump for mechanical pump fluid loops, apart from the design, relies on the performance of the closed impeller which is linked to the manufacturing process in terms of dimensional accuracy and the surface quality. Therefore, the activities of this paper were focused on defining the manufacturing process of a closed impeller using the additive manufacturing technology for mechanically pumped fluid loop (MPFL) systems in space applications. Different building orientations were studied to fabricate three closed impellers using selective laser melting technology and were subjected to dimensional accuracy and surface quality evaluations in order to identify the optimal building orientation. The material used for the closed impeller is Inconel 625. The results showed that both geometrical stability and roughness were improved as the building orientation increased, however, the blade thickness presented small deviations, close to imposed values. Finishing processes for inaccessible areas presented significant results in terms of roughness, nevertheless, the process can be further improved. Abrasive flow machining (AFM) post-processing operations have been considered and the results show major improvements in surface quality. Thus, important steps were made towards the development of complex structural components, consequently increasing the technological readiness level of the additive manufacturing process for space applications.

## 1. Introduction

The complexity of large space platforms requires thermal control systems able to maintain the functionality of spacecraft during the extreme temperature differences, specific to the space environment. There are several types of different active and passive systems which can be successfully applied in this domain, one of the most common active systems being the fluid loop system, where a liquid coolant is circulated in a closed loop under the action of a pump [1]. Compared to other systems, fluid loops are lighter and provide improved heat transfer characteristics than elements designed for conductive heat transfer, thus, they are frequently applied in state-of-the-art thermal control system designs. Therefore, large system integrators (LSIs) in Europe have shown interest in mechanically pumped loops (MPLs) for deep and manned space missions (transfer vehicles, orbiting stations, rovers, etc.) or telecommunication satellites [2].

The current market demands more centralized payloads, needing large heat transport capacity from the payload to the thermal radiators, but also high throughput satellites, with payload processors and active antennas requiring a high heat flux and/or high thermal stability. In addition, considering the human and robotic exploration strategy, in the framework of the International Habitat (I-HAB) or Orion MPCV—European Service Module, an active thermal control system is needed and the centrifugal pump can be equipped with an additively manufactured impeller. Several studies [2,3,4,5] show that MPL systems were dedicated only to the International Space Station (ISS) and deep space missions, until the year 2000, when the European Space Agency (ESA) began developing an MPL system for large telecommunication platforms (currently operating on a Russian GEO satellite) and a mechanically pumped fluid loop (MPFL) for a 3–6 kW single-phase MPL system. The results and knowledge gathered laid the groundwork for a large (7–20 kW) two-phase MPL development for the thermal control of telecommunication satellites on the Neosat platform. The development of a new and improved centrifugal pump for mechanical pump fluid loops helps in consolidating Europe’s position in the domain of spacecraft active thermal control systems and secures its independence from other states or space agencies.

Identifying a critical part in pump assembly, the manufacturing process could be improved in terms of performance, costs and time reduction. The closed impeller and the bearings are the main components that influence the pump performance. Having a low volume and a complex design that formative or subtractive methods are unable to produce, additive manufacturing (AM) technology seems to be best suited for the closed impeller. The manufacture of AM space components was performed by Brandão et al. [6] using a selective laser melting (SLM) process to determine the powder feedstock cross contamination of Ti-6Al-4V alloy, under different orientations. The results showed tensile strength values within the limits of the ASTM F2924 standard [7] for specimens built in the main directions on a process plate, while specimens built with respect to the building direction (Z) presented a larger variation of yield and tensile strength as compared to those built on the XY plane with a significantly lower fracture elongation. Moreover, X-ray computer tomography (CT) of all specimens confirms the W particle contamination, the origin being the cross contamination of the powder feedstock in the AM machine. This is expected to have a negative impact on other material properties, such as stress corrosion cracking and corrosion resistance.

The residual stress is a common aspect of interest of AM components. It can lead to part distortion, loss of geometric tolerance and delamination of layers during deposition, as well as deterioration of the fatigue performance and fracture resistance of the fabricated part, as presented by Deb Roya et al. [8]. To overcome this, stress relief treatment is effective for nickel-based super alloys [9]. On the other hand, care should be taken to optimize laser processing conditions to control residual stresses. For the SLM process, a laser scanning strategy that is used to melt the powder has a significant influence on the residual stresses that develop. Normally, the stresses are larger perpendicular to the scan direction than along the scan direction, and the influence of the process parameters was investigated by Gu et al. [10]. Poor dimensional accuracy is also generated by incorrect scaling/offset factors and is affected by part geometry, beam intensity and the density of the powder bed or SLM scan head/optics problems. It can be detected using X-ray computer tomography or dimensional accuracy analysis [11].

The hydraulic capacity and performance of AM centrifugal pump impellers was investigated by Fernandez et al. [12] using a different manufacturing technology, fused deposition modeling (FDM), and found that the inherent roughness of the AM process did not limit the head-flow curve results of the pump and, by using a chemical post-treatment, a more stable behavior in the high-flow operating range of the pump is ensured. The correlation between surface roughness, which depends mostly on surface temperatures achieved with as-built Inconel 625 SLM parts, and process parameters was found by Koutiria in [13] and Keller and Mendricky in [14]. Globally, Ra was shown to increase with lower scan speeds, to decrease with higher powers and to increase severely on down-skin sides for large building angles.

An important step in developing a closed impeller prototype using additive manufacturing technology was made by Sulzer [15], studying different orientations and support designs in order to avoid material deposition in inaccessible areas. Even if this AM component was not meant for space applications, it can be considered an excellent model of good practice and the results represent a step forward in the development of closed impellers for the space industry [16]. The Marshall Space Flight Center [16,17] designed and manufactured closed impellers, turbine components and housings for a liquid oxygen turbopump by direct metal laser sintering (DMLS) with an Inconel 718.

Additive manufacturing, especially SLM technology, represents an important part of the development of new prototypes and functional centrifugal pumps for mechanically pumped fluid loops for the space industry. A long-term goal is to use the AM closed impeller to equip a centrifugal pump for MPFL systems and to expand the use of AM technology in the development of new components for the space domain, like pumps, turbo pumps and other high-speed rotational equipment.

The objective of this paper is to give a preliminary validation of the advanced manufacturing process for an Inconel 625 closed impeller which has an important role in pump performance. The work is also focused on the advantages provided by selective laser melting technology, in terms of the quality of built parts, novel shapes and geometric complexity that is difficult or not achievable through conventional fabrication techniques, thereby widening the horizon of design in the space field by applying new technologies. The technical aspects that will be derived from this work will be used and optimized to manufacture an impeller to be integrated in the pump assembly for evaluating the hydraulic performance. 

## 2. Materials and Methods

### 2.1. Design Considerations

**Figure 1:**
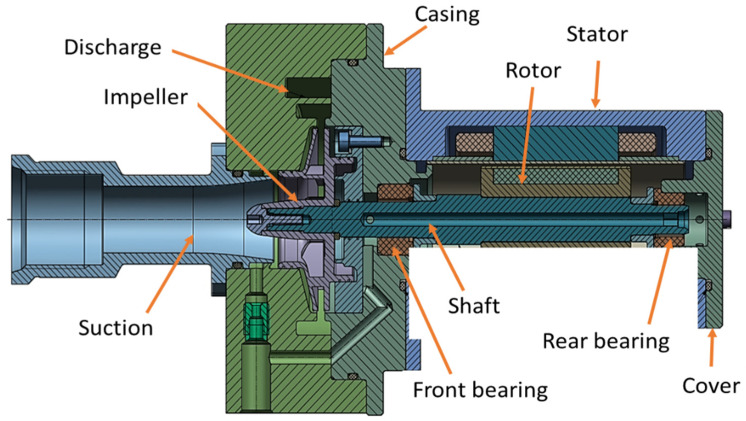
General pump architecture.

The primary challenge for AM space products is the fulfilment of qualification requirements and the guarantee that all part batches have the expected mechanical properties and the same high quality. The proposed solution is designed to fit a future pump assembly, following the general pump architecture presented in Figure 1. Technical specifications are presented in Table 1.

Although titanium and aluminium alloys are used in the fabrication of metallic components for space applications, Inconel 625 was used in the development of the closed impeller through SLM technology, as the objective of this study was to evaluate the AM process of such complex parts compared to conventional methods, in terms of manufacturing time and geometrical accuracy. The Inconel 625 powder (purchase from LPW Technology Ltd., Runcorn, UK) characteristics are presented in Table 2.

### 2.2. Design Concept

**Figure 2:**
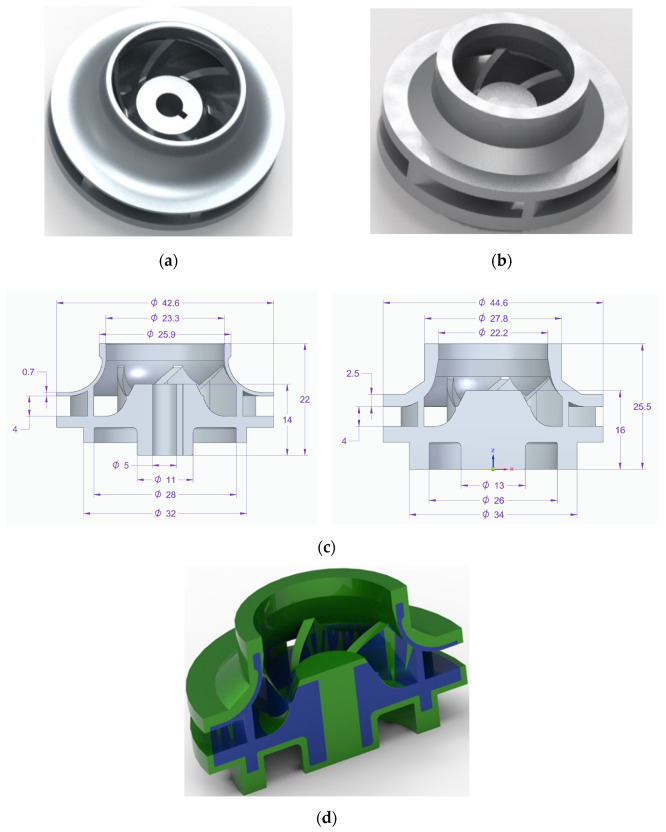
Representation of the initial closed impeller model (blue) and offset model (green) for the additive manufacturing (AM) process: (**a**) isometric view of the closed impeller model; (**b**) isometric view of the closed impeller model with offset material; (**c**) section view representation and dimensions of the two closed impellers (mm); (**d**) overlapping representation of the two impellers.

Using a pre-defined impeller blade profile and body, the computer-aided design (CAD) model for the closed impeller was obtained using Solid Edge (version 2019, Siemens PLM Software, Cologne, Germany), as presented in Figure 2a. Due to the recommendations and constraints specific to the AM process (minimum wall thickness, channels, holes and machining allowance), an offset on the external impeller surfaces was added to the design model that will be removed through mechanical post-processes. This ensures a rapid and controllable geometrical accuracy. The offset model was translated to a stereolithography (STL) model using the same software, with a conversion tolerance of 0.001 mm and a surface plane angle of 1°. For a better understanding, an overlapping representation of the two models is presented in Figure 2d. The overall estimated mass for the closed impeller after the AM process was 122 g, while for the finished one, the mass was estimated to be 60 g.

### 2.3. SLM Printing Process

**Figure 3:**
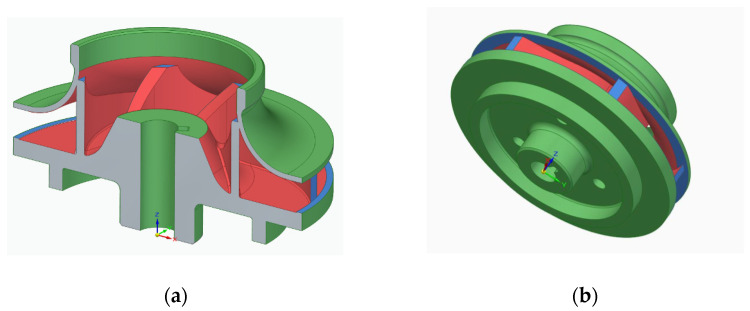
Support material deposition: red: impossible to remove; blue: difficult to machine; green: easy to remove: (**a**) closed impeller—section view and (**b**) closed impeller—isometric view.

The SLM process was carried out using a Lasertec 30SLM facility (DMG MORI, Bielefeld, Germany) owned by the Romanian Research and Development Institute for Gas Turbines (COMOTI), with a building volume of 300 mm × 300 mm × 300 mm. The manufacturing approach for the additive manufacturing technology must be in line with specific standards and the implication of new technology and special attention must be paid to the manufacturing process in terms of:

- impeller positioning and orientation in the building chamber to avoid, as much as possible, the deposition of support material between the blades which is impossible to remove, as indicated in Figure 3;
- material addition on the part’s exterior for post-processing operations to obtain a good roughness (from 0.8 to 1.6 Ra);
- printing parameters: layer thickness, laser power, scan strategy, focus, scanning speed.

To observe if the closed impeller is suitable for AM technology, a simplified geometry of the open impeller was considered as a first manufacturing trial, at the orientation of the Z axis (building direction). Closed impellers have shrouds on both sides of the blades, while open impellers have only one shroud opposite the impeller entrance. As they do not have any protective shroud, the open impellers tend to be weaker and are typically used for pumps that are not operated under significant constraints. No other tilting angles were considered for the open impeller as the support material would have been attached to the blades. The second manufacturing trial comprises the closed impeller in three orientations: one built at the orientation of the Z axis and other two built at B + 32° and B + 42°. The terminology for building orientations is in line with standard terminology for additive manufacturing [18]. Two aspects were considered when the orientations of the component were chosen, avoiding the attachment of the support structure to the blades and avoiding the overhanging of the shroud (self-supporting area). After a preliminary ANSYS (version 2020 R1, Ansys Inc., Canonsburg, PA, USA) simulation, these orientations were indicated as suitable for this application considering the conditions mentioned earlier.

Other printing angles were investigated and analysed with respect to geometrical stability. These angles were correlated with different printing parameters to obtain the best results in terms of geometrical stability and avoiding the overhanging area. These results, along with CT scans, internal surface finishing and balancing results, will be the subject of a follow-up paper on which we are presently working.

**Figure 4:**
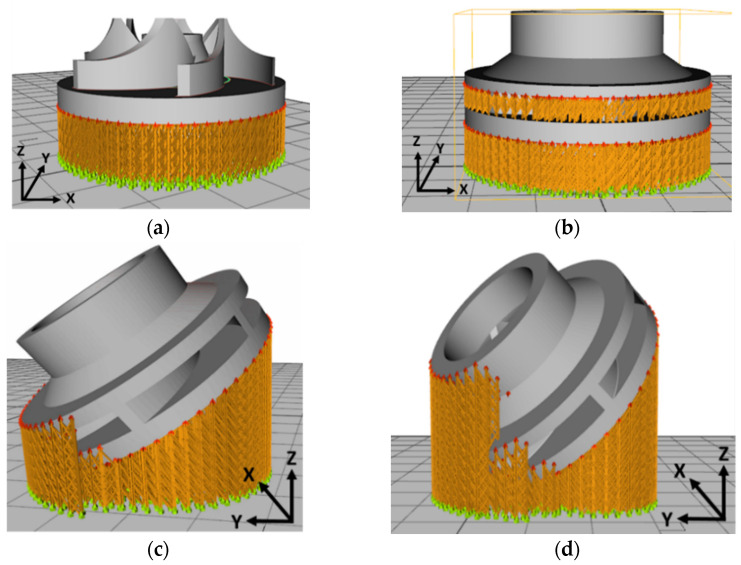
Building direction and support structure for the open and closed impeller models: (**a**) open impeller—built at orientation of Z axis (building direction); (**b**) closed impeller—built at orientation of Z axis (building direction); (**c**) closed impeller—built in orientation B + 32° (angled 32°); (**d**) closed impeller—built at orientation B + 45° (angled 45°).

For each building orientation, a set of two impellers was fabricated. Table 3 presents the process parameters used for the closed impeller fabrication as previously performed in the work done by Condruz et al. [19]. Figure 4 comprises the four impeller CAD models with simulated printing support material and printing parameters, using RDesigner (version 2018, Realizer GmbH, Borchen, Germany) software. 

### 2.4. Post-Processing Operations

The closed impellers were subjected to two post-processing methods, abrasive flow machining (AFM) for deburring and polishing the interior surface of the parts and classical machining for removing the offset material for the AM process. Before machining the external surfaces, the closed impellers were subjected to stress-relieving heat treatment using an electric oven of chamber type T_max_ = 1200 °C, with a volume of 140 L and power consumption of 13.5 kW.

The AFM process performed with the VECTOR 8 Abrasive Flow Machining (AFM, Remscheid, Germany) system is ideal for polishing and deburring, especially for complex internal surfaces, using chemically inactive or non-corrosive media to improve the surface finish and edge conditions. The abrasive particles in the media grind away rather than shear off the unwanted material. Turning operations follow the AFM process as the machining process of the closed impeller to reach its final dimensions, and as a method of cost-effective and in-house processing.

### 2.5. Dimensional Accuracy Analysis

Roughness was measured using a Mahr Surf PS10 instrument (Mahr GmbH, Gottingen, Germany) before and after post-processing operations for process validation. A length of measurement of 0.8 mm × 10 mm was considered with a 1.0 mm/s speed with respect to impeller dimensions. Dimensional accuracy analysis was performed using a 3D laser surface scanning ATOS Compact Scan 5M machine (GOM GmbH, Braunschweig, Germany), integrated with GOM software for scanning and inspection with 2 × 5 × 10^6^ pixels and measuring point distance between 0.017 mm and 0.481 mm.

The measurements were realized considering optical triangulation, photogrammetry and fringe projection methods. Due to the reflective nature of the parts, white markers (0.8 mm diameter) were applied and evenly distributed on each side of the impellers, after which each impeller was mounted on a rotary table. Multiple scans from various angles were done in order to scan the entire surface of the impeller. The obtained scanned model was overlapped with and aligned to the CAD model and normal deviations were calculated and represented by means of a colored spectrum. The standard deviations ranged within the acceptable tolerance of ±0.1 mm.

## 3. Results

### 3.1. Impeller Fabrication and Analysis

**Figure 5:**
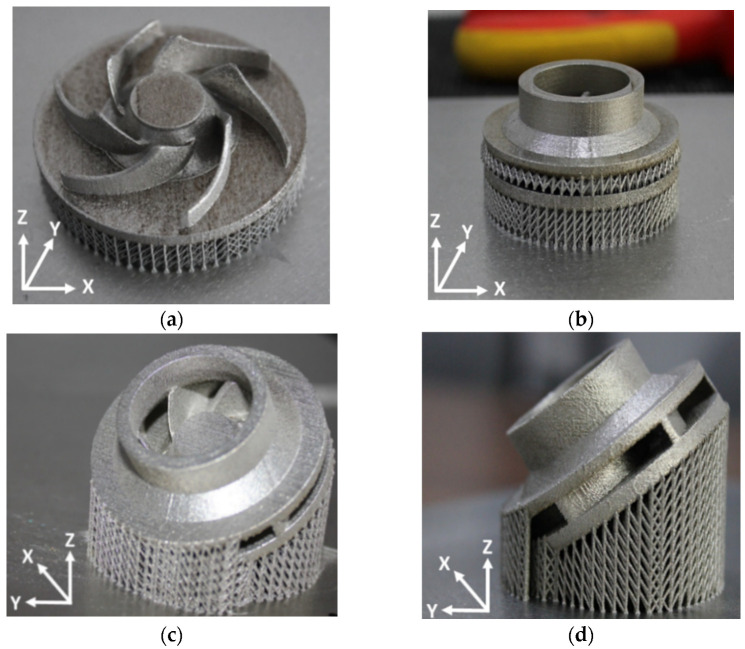
Open impeller and closed impellers built with different orientations on the building plate: (**a**) open impeller—built at orientation of Z axis (building direction); (**b**) closed impeller—built at orientation of Z axis (building direction); (**c**) closed impeller—built at orientation B + 32° (angled 32°); (**d**) Closed impeller—built at orientation B + 45° (angled 45°).

The as-printed parts on the building plate are illustrated in Figure 5. Considering the printing parameters shown in Table 3, the average printing time for one impeller was 14 h. Printing time may be influenced by the number of the pieces printed in the same process. The roughness of each printed impeller was measured over a distance of 10 mm with a 1 mm/s rate and the results are presented in Table 4. Roughness was measured only on the exterior surfaces of the shroud as the back of the impeller was machined to remove the support material.

It was observed that, by varying the tilting angle and due to the corresponding overhanging area, the shroud roughness has an increasing tendency. However, the exterior surface roughness is not important as it shall be machined to the final dimensions, but the dimensional stability of the overall impeller is critical. The closed impellers were then subjected to a stress-relieving cycle (stress relief at 870 °C with a heating rate of 5 °C/min for 60 min, air cooling and annealing at 1000 ± 5 °C for 60 min—oil quench). The heat treatment was applied prior to support structure removal by turning operations of the external surfaces.

#### 3.1.1. Evaluation of Exterior Dimensional Accuracy

**Figure 6:**
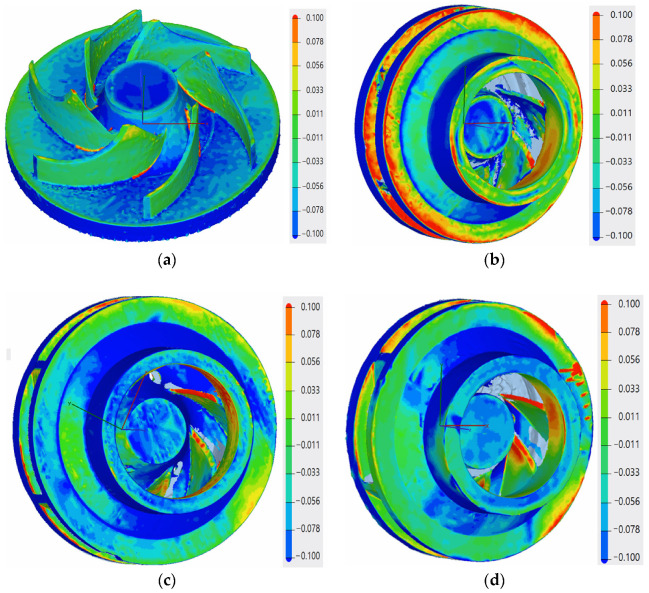
Exterior geometrical evaluation of the AM impellers: (**a**) evaluation of the open impeller for 0°; (**b**) evaluation of the closed impeller for 0°; (**c**) evaluation of the closed impeller for 32°; (**d**) evaluation of the closed impeller for 45°.

Prior to the geometrical evaluation of the as-printed parts, the support material was removed using classical machining (turning). The dimensional accuracy for the open impeller showed that the printing process follows the geometrical constraints of ± 0.1 mm on the blade positioning and tolerances, as presented in Figure 6a, with respect to the CAD model. For the three closed impellers, the variations of the component on the building platform at 0°, B + 32° and B + 45° showed an improvement in dimensional accuracy, as the average values are more consistent and closer to the imposed requirements. The blue shades on the back of the impellers were not considered deviations from the geometrical constraints as the turning process removed 0.1–0.2 mm of material during support structure removal. However, a profile deviation was observed on the exterior of the shroud top (maximum −0.235 mm) due to the printing angle, but as the offset for the SLM process was considered 1 mm, the resulting profile deviations from the printing process were considered acceptable.

#### 3.1.2. Evaluation of Interior Dimensional Accuracy

**Figure 7:**
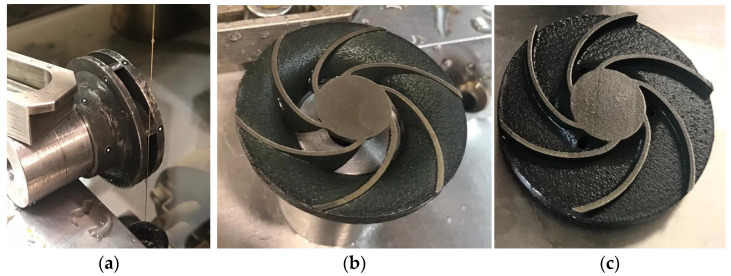
Electrical discharge machining (EDM) process of the closed impeller (45°): (**a**) EDM cutting process; (**b**) impeller shroud; (**c**) impeller disc.

**Figure 8:**
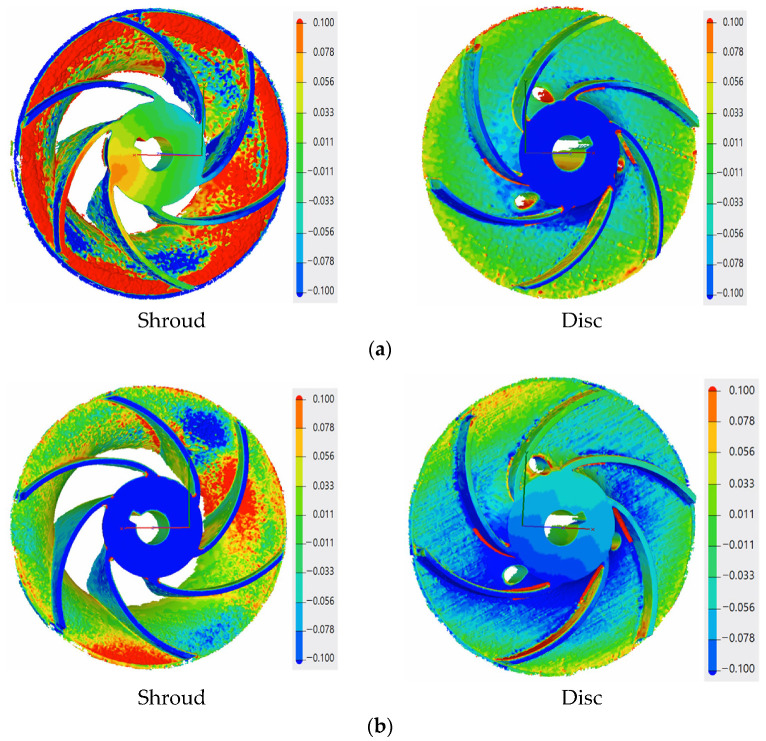

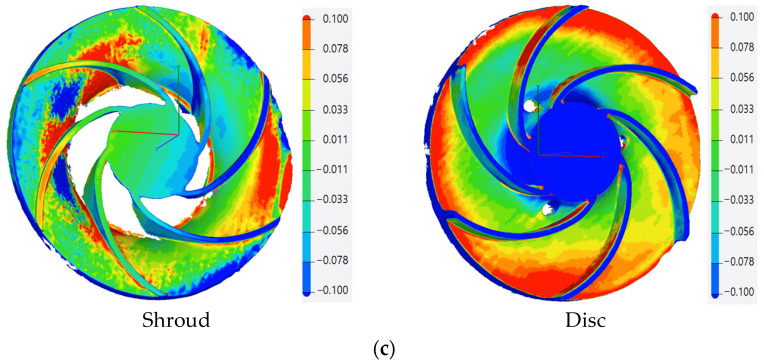
Geometrical evaluation of the AM closed impellers: (**a**) evaluation of the closed impeller for 0°; (**b**) evaluation of the closed impeller for 32°; (**c**) evaluation of the closed impeller for 45°.

In order to evaluate the interior deviations of the three closed impellers, each impeller was cut by electrical discharge machining (EDM), as a measure of precise cutting. A representation of the EDM process and impeller halves are presented in Figure 7, where the black color of the shroud and disc are due the heat treatment. The dimensional accuracy of each closed impeller is presented in Figure 8.

**Figure 9:**
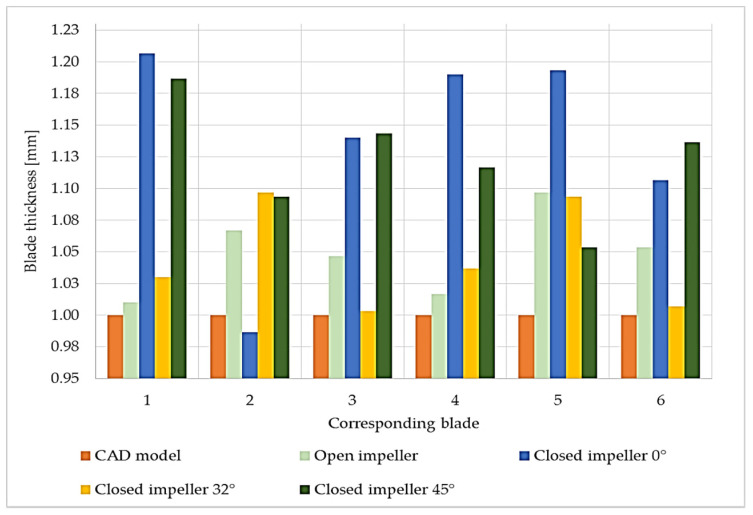
Blade thickness values measured using a caliper with an average of 3 values with respect to computer-aided design (CAD) model.

The red area on the shroud of the closed impeller printed at the orientation of the Z axis (building direction) shows the higher roughness of the part, as it is an overhanging area. The pressure side of the blades on the shroud part indicates a few profile deviations varying from −0.077 mm to +0.024 mm. However, these values are within the imposed tolerance of ±0.08 mm. For the closed impeller printed at a 32° orientation, the pressure side of the shroud blades presented a more stable geometrical accuracy compared to the suction side, but at the same time, the disc blades show an increase profile deviation for both the pressure and suction side (from −0.209 to +0.153 mm), higher than the imposed values. Further increasing the printing angle (45°) reduced the blades’ profile deviations from the imposed tolerance of ±0.08 mm. Nevertheless, due to the high tilting angle, the upper part of the impeller, namely, the foremost upper blade, presents a higher roughness due to its overhanging nature. Thus, a more intense red area appears on the suction side of the disc blade in Figure 8. Another analysis was made based on the blades’ thicknesses in order to verify if the printing angles also affect their dimensions, as this also represents an aspect that may influence the pump performance. Each blade was measured on both the shroud and the disc and a mean value is presented in Figure 9, as compared to the CAD model.

Although no blade profile deviations were observed for the open and closed impellers printed at the orientation of the Z axis (building direction), their blade thicknesses were affected, as can be seen from Figure 9. Although the open impeller presents acceptable thickness variations for all six blades (maximum ±0.1 mm), the closed impeller thickness variations are higher and are not compliant with the requirements. For the closed impeller printed at 32°, a more accurate and uniform thickness distribution was recorded, however, its blades’ profile deviations were higher than the imposed tolerances (Figure 8). For the third closed impeller, a more stable relationship was found between the tilting angle, profile deviation and blade thickness as compared with the two closed impellers printed at 0° and 32°. By varying the finishing process parameters, a more constant blade thickness can be obtained, but in the present case study, the main objective was to identify the most suitable printing angle in terms of geometrical evaluation. 

### 3.2. Post-Processing Evaluation

**Figure 10:**
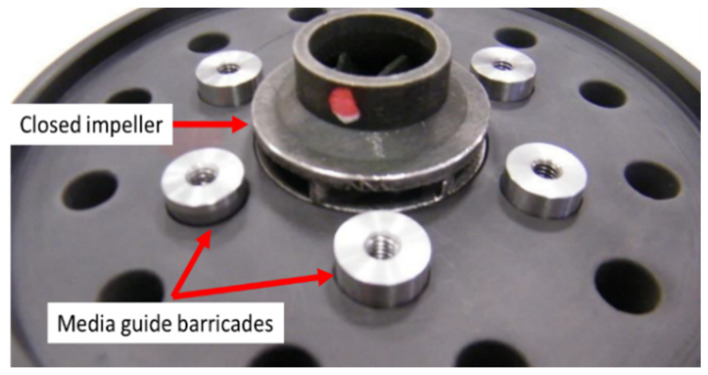
Abrasive flow machining (AFM) tooling dedicated to small AM closed impellers.

For the AFM process (performed at Extrude Hone GmbH, Holzgünz, Germany), a tooling was required to guide the media flow through the closed impeller flow channel and towards the blade tail, to ensure a uniform and constant surface roughness, as represented in Figure 10. For a trial procedure, only one closed impeller was tested and evaluated, printed at 45°. Process parameters are presented in Table 5.

**Figure 11:**
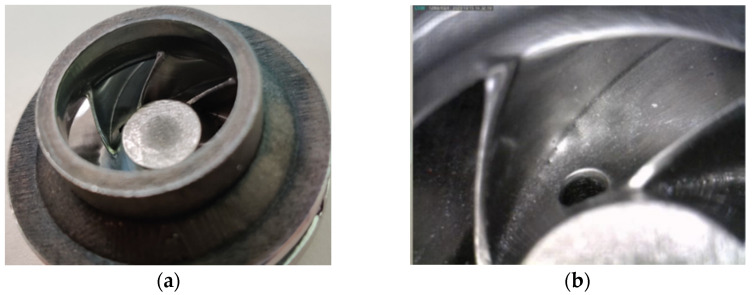
Images presenting the (**a**) closed impeller after AFM process and (**b**) close view of the finishing process.

After a visual inspection, the AFM finishing process showed good results in terms of surface roughness improvement. However, small areas at the suction side of each blade tail remained unfinished. This was a result of the impeller blades’ geometry which does not guide the media along the full extent of the blade. A method to overcome this problem is to use different medium guide barricades and a different process parameter and will be investigated in a future research. The finished closed impeller is presented in Figure 11.

**Figure 12:**
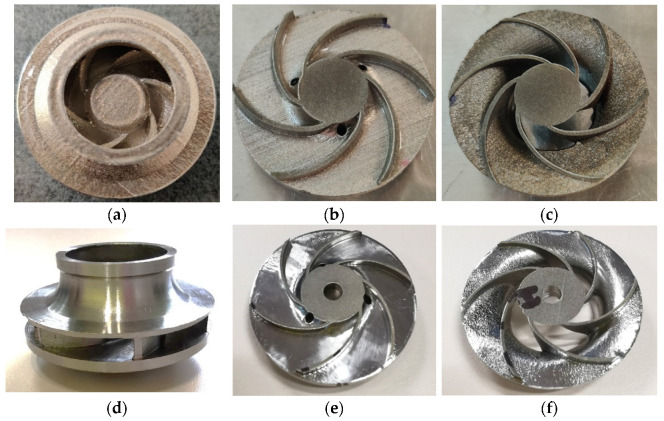
Post-processed (turned) closed impeller: (**a**) as-printed closed impeller; (**b**) as-printed disc half; (**c**) As-printed shroud half; (**d**) finished closed impeller; (**e**) finished disc half; (**f**) finished shroud half.

**Figure 13:**
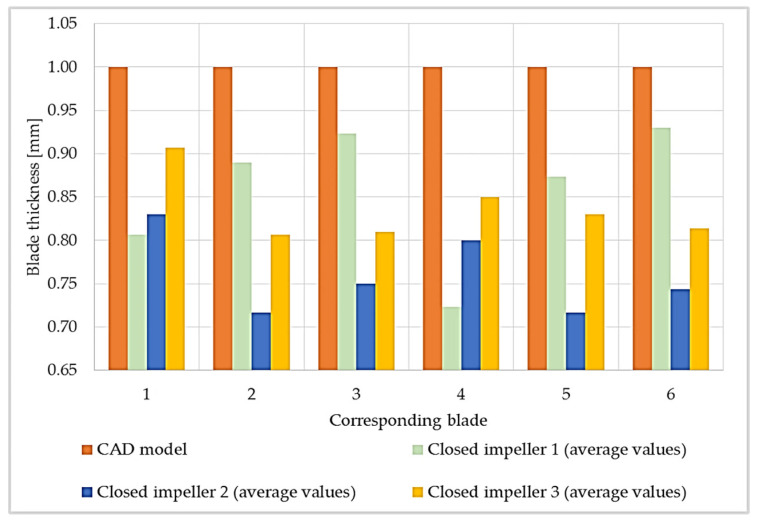
Blade thickness values measured using a caliper with an average of 3 values after AFM process values with respect to CAD model.

The AFM finished part was machined to its final dimensions, using turning operations, and a mean roughness value of Ra 0.6 μm was found on the closed impeller exterior. Before cutting the closed impeller to verify if the AFM process affects the blade thickness, the weight was verified and a mass loss of 2.70 g was found. This is important information for AFM process improvement and moreover for the balancing of the closed impeller, as a final verification step. The impeller was cut by EDM and each blade was measured at three points using manual calibrated equipment and the average values were calculated. Figure 12 presents a comparison between the closed impeller before and after both finishing operations and the two halves after EDM cutting. The blade thickness variation after the AFM process is presented in Figure 13 for three closed impellers. A maximum of ±0.1 mm thickness variation is allowed.

As can be observed from both Figure 12 and Figure 13, the AFM process affects the blade thickness. This is somewhat predictable as the medium glides over the pressure and suction side of the blades. However, changing the media and parameters could give a more uniform distribution of the media over the blade profile and consequently the blades thickness will be less affected. Another option is to optimize the tooling in such a way as to remove a more uniform quantity of material.

A roughness evaluation was also performed for the two halves of the impeller and a mean value for the shroud was found at Ra 3.85 μm and for the disc at Ra 0.66 μm, as compared to as-printed values of Ra 6.480–8.233 μm. Although the AFM process succeeded in reducing the roughness of the closed impeller interior, the shroud surface was not completely polished due to its geometry. As mentioned before, by changing the media and the parameters, the overall roughness can be reduced greatly. 

## 4. Discussion

An important step was made towards the development of new prototypes and functional centrifugal pumps, as the main objective of this study was to analyze the potential application of SLM technology in the fabrication of small centrifugal closed impellers for MPFL pumps. 

Due to the high interest in using AM technology for space components, the results achieved in the current paper present a complete manufacturing process with respect to closed components with small dimensions suitable for space applications. Additionally, a new approach for the post-processing of complex geometries, as closed components, has been studied. 

One of the main concerns in developing such small, closed parts represents the deposition of support material in areas where it is impossible to remove it. Therefore, three building directions were investigated under this study, 0°, B + 32° and B + 45°, to evaluate the AM process of such components. After a simulation of the deposition of support material, the closed impellers, along with one open impeller, were fabricated and analysed. As is already known, the roughness of AM parts is usually higher, but after one or more post-processing operations, the overall roughness can be reduced greatly. 

For all three closed impellers, it was observed that by increasing the printing angle, a better dimensional stability is obtained, for both the exterior regions as well as for the blade surface accuracy. However, attention must be paid to the overhanging areas which affect both the roughness and the dimensional stability of the parts. Even though the dimensional stability was increased when increasing the printing angle, the blades did not present the same trend in terms of thickness. The part printed using the 0° orientation presented the most thickness deviations from the CAD model. The 32° and 45° building angles presented the least thickness variations as compared to the CAD model, and the one printed at 32° is within the imposed limits, but with higher profile deviations. As the blade thicknesses in the impeller printed at 45° are close to the imposed limits, but a more stable profile was observed, a compromise can be made.

Another important step in the development of AM parts, and especially for the space industry, is the post-processing operations. AFM was selected as the most suitable process for polishing the interior areas of such small components. The first AFM trial shows a great improvement in surface finishing, for both the blade pressure and suction side and for the flow channel. However, small difficulties were identified at the blades’ tails due to their geometry, and to their thickness. Moreover, the AFM process shows a great roughness improvement for the impeller interior, especially in the disc region, yet, due to the impeller’s geometry, the shroud was not polished sufficiently.

To assess the identified problems in terms of dimensional stability, roughness and thicknesses, future research should include investigations of different angle orientations for the same design model, as well as different AFM process parameters. Moreover, to enhance the AFM finishing operation, a process simulation can be performed to anticipate the area that will be most rapidly worn out. Thus, other media and parameters are under study as well.

It should be mentioned that the selected material for this study is compatible with the pump working fluid and, in the next development step, the hydraulic performances of the pump will be investigated when equipped with an identical impeller in terms of material, SLM manufacturing process and post-processing.

## 5. Conclusions

The study advances the knowledge towards increasing the technological readiness level of the additive manufacturing of complex metallic components without internal support structures for space applications. Additionally, a customized approach for post-processing the inner surfaces of complex geometries, such as closed components, has been studied and presented.

Within this work, the fabrication process of a closed impeller for MPFL systems by means of additive manufacturing was studied. SLM technology was selected as the AM process using IN615 alloy. One open impeller and three closed impellers were printed at different orientations, to evaluate their geometrical stability, in terms of dimensional accuracy for both as-printed parts and blade deviations. It was concluded that a printing orientation of B + 45° presents the best geometrical correlation between blade deviations and thicknesses. The 45° orientation was a preliminary set point for manufacturing a small closed impeller with a complex geometry without the deposition of support material in areas inside the closed impeller that are impossible to remove. Following this, two post-processing operations were considered for the 45° AM closed impeller, to evaluate its final dimensions. Both the AFM and turning operations showed great results in terms of roughness improvement and dimensional stability, however, the AFM process requires a more adaptable medium and process parameters in order to overcome problems and improve the results. 

To quantify the printing process and functionality of the AM closed impeller, a more extensive study was done considering non-destructive tests (computer tomography scans) and an optimized AFM finishing process and shall be presented in a follow-up paper. 

The results obtained from this study offer an important step in defining and developing an AM component with complex geometry, in order to increase the technological readiness level for the development and qualification of additively manufactured closed centrifugal impellers for space applications.

## Figures and Tables

**Table 1:** Pump technical specifications.

Pump Type	Centrifugal, Canned Motor, Constant Speed (Settable), Brushless
Flow rate	0.1667–0.2639 (kg/s) HFE7200
Inlet pressure	150,000–300,000 (Pa) without any impeller cavitation
Pressure rise	200,000–400,000 (Pa)
Operating life	Orbit life shall be greater than 15 years (goal)
Operating environment temperature range	253.1500–323.1500 K
Non-operating environment temperature range	243.1500–333.1500 K
Impeller	The impeller shall be suspended by hydraulic thrust bearing

**Table 2:** Inconel 625 powder characteristics: (**a**) powder dimensions; (**b**) powder flowability.

(**a**)	
Particle size	15–45 µm
Particle size distribution	D10 = 20 ± 2 μm; D50 = 30 ± 5 μm; D90 = 45 ± 5 μm
(**b**)	
Hausner ratio	<1.25
Angle of response	<40°
Relative density	Minimum 99.5% (with no contour)

**Table 3:** Selective laser melting (SLM) process parameters [19].

The Process Parameters Were the Same for All Printed Parts	
Laser power (W)	250
Scanning speed (mm/s)	750
Layer thickness (µm)	40

**Table 4:** Average roughness values of as-printed impellers.

Values	Open Impeller		Closed Impeller: 0°		Closed Impeller: 32°		Closed Impeller: 45°	
	Ra	Rz	Ra	Rz	Ra	Rz	Ra	Rz
Average (μm)	6.480	43.581	6.689	47.606	7.646	68.745	8.233	70.989
Std. dev.	0.051	0.609	0.332	2.959	0.338	2.21	0.25	0.491

Ra: roughness average, Rz: average maximum height of the profile and Std. dev.: standard deviation.

**Table 5:** AFM process parameters.

Medium	Pressure	Volume	Cycles
649 Z1 BC	35 (bar)	0.00819 (m^3^)	10

## Data Availability

The data presented in this study are available on request from the corresponding author. Due to the contract agreement between COMOTI and funding agency, the research activities and data presented in this paper will be presented in an Executive Summary Report and will be available for public use, after project closure.

## References

[1] Lam T.T., Birur G.C., Bhandari P., Gilmore D.G. (2002). Spacecraft Thermal Control Handbook, Volume I: Fundamentals Technologies, Chapter 12: Pumped Fluid Loops.

[2] van Es J., van Gerner H.J., van Benthem R.C., Lapensee S., Schwaller D. Component Developments in Europe for Mechanically Pumped Loop Systems (MPLs) for Cooling Applications in Space. Proceedings of the 46th International Conference on Environmental Systems, ICES-2016-196.

[3] Raetz J.E., Dominick J. (1992). Space Station External thermal control system design and operational overview. SAE Tech. Pap. Ser..

[4] Benthem R., Elst J., Bleuler R., Tjiptahardja TDutta P. (2009). Development of a Mechanically Pumped Fluid Loop for 3 to 6 kW Payload Cooling. SAE Tech. Pap. Ser..

[5] Merino A.S., Hugon J., Cailloce Y., Michard F., Tjiptahardja T., Larue de Tournemine A., Laporte C. Development of a Two-Phase Mechanically Pumped Loop (2ΦMPL) for the Thermal Control of Telecommunication Satellites. Proceedings of the International Two-Phase Thermal Control Technology Workshop, ESTEC.

[6] Brandão A.D., Gerard R., Gumpinger J., Beretta S., Makaya A., Pambaguian L. (2017). Ghidini, Challenges in Additive Manufacturing of Space Parts: Powder Feedstock Cross-Contamination and Its Impact on End Products T. Materials.

[7] ASTM F2924-14 (2014). Standard Specification for Additive Manufacturing Titanium-6 Aluminum-4 Vanadium with Powder Bed Fusion.

[8] DebRoy T., Wei H.L., Zuback J.S., Mukherjee T., Elmer J.W., Milewski J.O., Beese A.M., Wilson-Heid A., De A., Zhang W. (2018). Additive manufacturing of metallic components—Process, structure and properties. Prog. Mater. Sci..

[9] Bhargava A.K., Banerjee M.K. (2017). Heat-Treating Copper and Nickel Alloys. Compr. Mater. Finish..

[10] Gu D.D., Meiners W., Wissenbach K., Poprawe R. (2012). Laser additive manufacturing of metallic components: Materials, processes and mechanisms. Int. Mater. Rev..

[11] Toma A., Condruz R., Carlanescu R., Daniel I. (2020). A Mini-review on Non-destructive Techniques for Additive Manufactured Metal Parts. AIP Conf. Proc..

[12] Fernandez S., Jimenez M., Porras J., Romero L., Espinosa M.M., Dominguez M. (2016). Additive Manufacturing and Performance of Functional Hydraulic Pump Impellers in Fused Deposition Modeling Technology. J. Mech. Des..

[13] Koutiria I., Pessardb E., Peyrea P., Amloua O., de Terrisa T. (2018). Influence of SLM process parameters on the surface finish, porosity rate and fatigue behavior of as-built Inconel 625 parts. J. Mater. Process. Technol..

[14] Keller P., Mendricky R. (2015). Parameters influencing the precision of SLM production. MM Sci. J..

[15] Huber M., Hartmann M., Ess J., Loeffel P.D., Kränzler T., Rettberg R. Process method for manufacturing impellers by Selective Laser Melting (SLM). Proceedings of the International Conference on Additive Manufacturing in Products and Applications (AMPA) ETH.

[16] Derek O. Applying Additive Manufacturing to a New Liquid Oxygen Turbopump Design. Proceedings of the Additive Manufacturing for Propulsion Applications Technical Interchange Meeting/JANNAF.

[17] Laura Russart, 3D Printed Rocket Engine. https://fathommfg.com/gpi-prototype-builds-3d-printed-inconel-718-rocket-engine-for-seds-ucsd.

[18] (2013). ISO/ASTM 52921:2013 Standard Terminology for Additive Manufacturing—Coordinate Systems and Test Methodologies.

[19] Condruz M.R., Matache G., Paraschiv A., Frigioescu T.F., Badea T. (2020). Microstructural and Tensile Properties Anisotropy of Selective Laser Melting Manufactured IN 625. Materials.

